# Evolution of *Helicobacter pylori* Resistance to Antibiotics: A Topic of Increasing Concern

**DOI:** 10.3390/antibiotics12020332

**Published:** 2023-02-04

**Authors:** Lyudmila Boyanova, Petyo Hadzhiyski, Raina Gergova, Rumyana Markovska

**Affiliations:** 1 Department of Medical Microbiology, Medical Faculty, Medical University of Sofia, Zdrave str. 2, 1431 Sofia, Bulgaria; 2 Specialized Hospital for Active Pediatric Treatment, Medical University of Sofia, “Acad. Ivan Evstatiev Geshov” blvd., 1606 Sofia, Bulgaria

**Keywords:** *Helicobacter pylori*, evolution, increase, antibiotic, resistance, amoxicillin, metronidazole, clarithromycin, tetracycline, levofloxacin

## Abstract

Antibiotic resistance among *Helicobacter pylori* strains is the major cause of eradication failure. Resistance prevalence is dynamic and can greatly vary among countries over the years. We revealed *H. pylori* resistance trends for five antibiotics in 14 countries through articles predominantly published in 2018–2022, since the latest data can best show the most recent trends in resistance evolution. Amoxicillin resistance generally exhibited no evolution, yet it increased in Bulgaria, Iran, China, and Vietnam. Metronidazole resistance exhibited different trends, including an increase, a decrease and no evolution in six, three, and five studies, respectively. Clarithromycin resistance increased in Australia, Belgium, Bulgaria, Italy, Iran, and Taiwan, but remained stable in France, Spain, Russia, China, Chile, and Colombia. Tetracycline resistance was low and stable except in Iran. Levofloxacin resistance increased in four European and six other countries/regions, without significant increases in France, Spain, and Chile. In Chile, triple resistance also increased. In countries such as France and Spain, resistance to most antibiotics was stabilized, while in Bulgaria, Belgium, Iran and Taiwan, resistance to three or more agents was reported. Use of non-recommended regimens, national antibiotic consumption, patient’s compliance, host factors, strain virulence, migrations, and azithromycin overuse during the COVID-19 pandemic can influence resistance evolution. New drugs, eradication regimens and diagnostic methods, such as next-generation sequencing can improve *H. pylori* infection control.

## 1. Introduction

*Helicobacter pylori* is a Gram-negative, spiral-shaped and microaerophilic bacterial species closely adapted to humans [1]. It has great medical importance as one of the most frequent causative agents of human infections. In the systematic review and meta-analysis of Zamani et al. [2] encompassing 73 countries worldwide, it has been emphasized that *H. pylori* infects >44% of the global human population, including >1/2 of the inhabitants of developing countries and >1/3 of those in developed countries.

These bacteria cause chronic gastritis, gastric and duodenal ulcers, gastric cancer or mucosa-associated lymphoid tissue (MALT) lymphoma [3], and although the infection is asymptomatic in 80–90% of the infected subjects, it carries some risks of severe diseases. Gastric cancer is the second leading cause of cancer-related mortality worldwide, and *H. pylori* has been recognized as one of the most potent carcinogens by the World Health Organization (WHO) [3].

*H. pylori* infection is most often acquired in childhood and becomes chronic, frequently lifelong, if not successfully eradicated [4]. *H. pylori* eradication (negative results from repeated tests for *H. pylori* detection at least 4 weeks after treatment) is curative, however it has been constantly decreasing over the years, mainly due to bacterial resistance [5]. Different composite regimens have been used to eradicate the infection; they have usually included a proton pump inhibitor (PPI) or (more recently) vonoprazan to increase gastric pH, plus two or three antibacterial agents, sometimes with the addition of bismuth preparations [5,6,7]. Amoxicillin, metronidazole, clarithromycin, tetracycline and levofloxacin are the most frequent antibiotics used in different combinations in eradication regimens.

Despite the complex treatment regimens, antibiotic resistance is the major cause of eradication failure. *H. pylori* resistance to antibiotics is most often due to chromosomally encoded mutations. However, it can also appear due to efflux systems, membrane permeability changes and biofilm formation [8,9,10]. Moreover, the lack of *H. pylori* susceptibility testing in most laboratories, as well as side effects of eradication regimens, can additionally hamper eradication success [7].

The aim of the present review was to determine the recent evolution of *H. pylori* resistance to five antibiotics of choice in eradication regimens in different countries worldwide.

## 2. Methods

We reviewed some recent data on the dynamics of *H. pylori* resistance to antibiotics that can cause *H. pylori* treatment failure. We considered data from recent studies providing details on patients’ numbers and characteristics, study methods, breakpoints for resistance, and resistance rates in different years. Data from recent years were included as they can best show the most recent trends in resistance evolution. For this purpose, we evaluated *H. pylori* resistance evolution by searching PubMed, Science Direct, and Google Scholar for publications with the following keywords or word combinations in the title or abstract: “*Helicobacter pylori*”, “*H. pylori*”, “antibiotic”, “antibacterial”, “resistance”, “primary”, “secondary”, “evolution”, “prevalence”, “rate”, “trend”, and “increase”. We included publications published since 2018, with rare exceptions.

**Figure 1:**
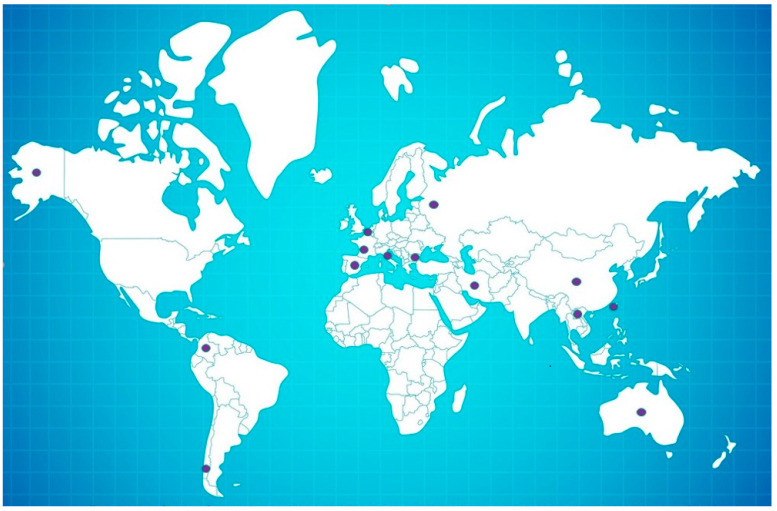
The countries from which data are included. The map is taken from the website: https://www.freepik.com/free-vector/earth-map-linear-composition_9386670.htm (accessed on 10 January 2023). The countries are marked with dots by the authors.

Data from several review articles and from 14 countries, including Australia, Belgium, Bulgaria, Chile, China, Colombia, France, Italy, Iran, Russia, Spain, Taiwan, Vietnam and USA were included and discussed (Figure 1).

Most articles were in English and only few were in Spanish. The most commonly used susceptibility testing method in the studies was an E test, while the agar dilution method, disk diffusion method and molecular tests were less frequently applied. The most commonly used breakpoints for resistance were those of EUCAST, although some studies used CLSI breakpoints or others specified by the authors (see below).

We also included our recent findings on *H. pylori* resistance in strains from consecutive Bulgarian patients with gastroduodenal diseases during the first study period (2007–2014 for amoxicillin and 2010–2015 for the other antibiotics) and the second period (2015–2021 for amoxicillin and 2016–2022 for the other antibiotics) [11,12], (Boyanova, this study). Most data (83.6%) in our unpublished study are from 2018 to 2022.

## 3. Results and Discussion

### 3.1. Evolution of Antibiotic Resistance in H. pylori over the Years

#### 3.1.1. Amoxicillin

Amoxicillin is a beta-lactam antibiotic that binds to penicillin-binding proteins (PBPs) in bacterial periplasm and affects transpeptidase activity for cross-linking of peptidoglycan molecules, thereby impeding peptidoglycan synthesis and *H. pylori* growth [13]. Amoxicillin is a frequently used antibiotic for *H. pylori* eradication in various regimens, such as: triple amoxicillin-based regimens, concomitant quadruple regimen, high-dose dual therapy, sequential therapy, hybrid therapy, as well as in some vonoprazan-based regimes [14,15,16,17,18].

Amoxicillin resistance in *H. pylori* usually results from mutations leading to diminished binding of the agent to penicillin-binding protein PBP1A, and mutations in PBP2 or PBP3 may additionally increase the resistance [10]. In 2006–2016, *H. pylori* resistance to amoxicillin was rare (0–<10%) in the WHO regions [19].

In the present review, no evolution of *H. pylori* resistance to amoxicillin was detected in most countries, such as Belgium, France, Russia, Australia, USA, Colombia, Chile and Taiwan [20,21,22,23,24,25,26,27,28,29,30,31,32]. It is noteworthy that no increase in amoxicillin resistance was observed in studies of treated patients in France (0.0% in 2014 and 2018, and 0.8% in 2016) and Taiwan (after one and two failed eradication attempts) [22,32].

However, a significant increase in overall amoxicillin resistance was detected in consecutive Bulgarian patients (2.1-fold rise from 2007 to 2014 to 2015 to 2021), in China (6-fold increase from 1998 to 1999 to 2016 to 2017) and Iran (4-fold increase from 1999 to 2000 to 2017 to 2019), and in primary *H. pylori* resistance in Vietnam (from 2013 to 2015 to 2019 to 2020) [11,33,34,35] (Table 1).

The growing amoxicillin resistance in Vietnam was explained by the frequent use of amoxicillin/clavulanic acid for various infections in the country [35]. As for Bulgaria, consumption of antibiotics has been high and increasing. From 2012 to 2021, while a significant decline in the consumption of J01 antibiotics (antibacterials for systemic use) was detected by the European Union/European Economic Area population-weighted mean overall, a significant increase was observed in Bulgaria [36].

Although the evolution of *H. pylori* resistance can be accounted for using the same resistance breakpoints in the same longitudinal study, the percentage of resistance depends on both the methods and the resistance breakpoints used [12,37]. It should be mentioned that the EUCAST breakpoints for amoxicillin resistance are lower (>0.12 mg/L) than those used in some studies, such as in China [33], and that some authors used a disk diffusion method to assess resistance [31].

Amoxicillin resistance rates have remained low over time (0–<2%) in countries such as Belgium, France, Australia and Colombia [20,21,22,27,29,30]. However, the present results show the need to monitor the dynamics of the resistance over prolonged periods of time due to its increase in some countries. Moreover, amoxicillin resistance was found to be an independent risk factor of *H. pylori* eradication failure when using clarithromycin-based triple therapy [37].

#### 3.1.2. Metronidazole

Metronidazole is a prodrug which becomes active when it is activated by the reduction of its nitro group, and thus causes bacterial DNA damage [38,39]. Metronidazole and sometimes tinidazole have comparable activity and are used in different *H. pylori* eradication regimens, such as triple regimens, bismuth quadruple therapy, sequential therapy and concomitant quadruple regimen [15,16].

Metronidazole resistance in *H. pylori* can result from different mechanisms, such as decreased prodrug activation by mutations in *rdxA* and *frxA* genes, mutations leading to RecA DNA repair upregulation and other mechanisms [10,40].

The systematic review and meta-analysis of Savoldi et al. [19] revealed that in 2006–2016, primary metronidazole resistance rates most often ranged from 23 to 56% worldwide, while secondary resistance reached >62% in the Eastern Mediterranean Region and the Western Pacific Region.

Recent data showed different trends in metronidazole resistance rates in *H. pylori* over time. An increase in resistance was observed in six studies, a decrease was found in three reports, and no evolution was detected in five studies.

Rising metronidazole resistance rates were found in untreated patients from Belgium, France and Taiwan and in consecutive patients from Bulgaria, Italy and Russia [12,20,21,22,26,32,41] (Boyanova, this study). In Taiwan, metronidazole resistance in *H. pylori* increased in both untreated and treated adults [32] (Table 2).

An increase in 2010/13, followed by a plateau in 2013/16, was found in one of the two Italian studies [41].

Decrease in *H. pylori* metronidazole resistance was also detected, although less frequently. Resistance to metronidazole diminished in Chile and Spain, as well as in the study of nine European countries [24,31,42]. No significant evolution has been reported in an Italian study, in Australia, Colombia, Iran, and in Spanish children [25,27,29,30,33,34,43] (Table 2).

However, it is a concern that high *H. pylori* metronidazole resistance was present in >75% of consecutive Colombian adults in 2009 and 2015, in consecutive Chinese adult patients in 1998–1999 and 2016–2017, as well as in treated patients in France (in 2014–2018) and Taiwan (in 2019) [22,29,30,32,33].

One of the reasons for the increase in the resistance may be the wide use of nitroimidazoles in some countries to treat *H. pylori* and other infections, such as dental and oral infections, anaerobic infections, bacterial vaginosis, pelvic inflammatory disease and parasitic diseases. In Belgium, the rise in metronidazole resistance was probably linked to the growing number of immigrants from Africa (by 18.7%) in the period 2010–2015 [20,21].

Importantly, increasing the therapeutic dose of metronidazole to 1500 mg and treatment duration up to 14 days in the bismuth quadruple regimen can either partially or completely overcome metronidazole resistance of *H. pylori* [7,22,23]. It is also important that the accuracy of susceptibility testing results for metronidazole depends on the appropriate redox potential of the media used [23].

#### 3.1.3. Clarithromycin

Among all macrolides, clarithromycin is the most important agent for eradication therapy of *H. pylori* infection. Clarithromycin is a bacteriostatic antibiotic, acting by inhibiting bacterial protein synthesis through reversible binding to the 50S ribosomal subunit in the 23S rRNA gene of *H. pylori* [10].

Clarithromycin was included in different eradication regimens such as triple regimens, concomitant quadruple therapy and sequential therapy [15,16,17,18].

Overall, primary clarithromycin resistance in *H. pylori* in 2006–2016 was >15% in the European region, reaching ≥33% in the Eastern Mediterranean Region and Western Pacific Region, and secondary resistance (15 to 67%) to the agent was found in the WHO regions [19].

Clarithromycin resistance was most often linked to A2142C, A2142G and A2143G mutations in V domain of 23S rRNA, and some minor mutations outside the domain [10].

Recent data showed an increase in clarithromycin resistance in *H. pylori*, in patients from numerous countries, such as untreated patients in Belgium, consecutive patients in Australia, Bulgaria, Italy, Iran and South-East Asia, and in both untreated and treated patients in Taiwan [12,19,20,21,27,32,34,41,43] (Boyanova, this study) (Table 3). Between 1998 and 2017, *H. pylori* resistance to clarithromycin in Australia increased by 3.7% annually [27].

No increase in primary *H. pylori* resistance and decrease in post-treatment resistance were found in France [22]. Many other studies, such as those in Chile, Colombia, Russia and Spain (two studies), have also reported no evolution of clarithromycin resistance rates [24,25,26,29,30,31,33,42].

As stated by Megraud et al. [23], macrolide consumption (of intermediate-acting agents such as clarithromycin, and long-acting agents such as azithromycin) in the community can strongly affect the primary clarithromycin resistance in *H. pylori* several years later. The lack of clarithromycin resistance evolution in France can be due to the decrease of macrolide consumption (by >46%) from 2000 to 2015, and its stability during the last years [23]. The decrease in secondary clarithromycin resistance in French patients has been explained by increasing the use of recommended quadruple therapies as a first-line regimen [22].

By contrast, the increase in overall clarithromycin resistance in *H. pylori* in Bulgaria can be associated with high macrolide, lincosamide and streptogramin (J01F) consumption (5.5 DDD per 1000 inhabitants per day) in 2021, compared to that of other European countries [36].

Several other factors can be associated with the increase in *H. pylori* clarithromycin resistance over the years. Apart from *H. pylori*-associated diseases, macrolides are also used to treat upper and lower respiratory tract infections, and sexually transmitted infections. *H. pylori* clarithromycin resistance was >15%, with the exception of studies in Russia and Colombia [26,29,30], and the highest resistance rates (50% or more) were found in consecutive Chinese adults, treated French adults, Spanish children, and treated adults in Taiwan [22,25,32,33]. Frequent use of a triple clarithromycin-based regimen in countries with high resistance to the agent can contribute to the increase in *H. pylori* resistance to clarithromycin. In countries where resistance prevalence to clarithromycin is high or increasing over time, the use of clarithromycin-based triple therapy may only be appropriate if susceptibility testing is performed, and isolates are found to be susceptible to the agent [29].

Overuse or misuse of azithromycin has increased since the beginning of the COVID-19 pandemic and can also influence *H. pylori* macrolide resistance [44]. In Australia, migration from countries with high resistance prevalence or exposure to macrolides in food has been suggested as a factor contributing to the increase in *H. pylori* clarithromycin resistance [27].

Detection of clarithromycin resistance before using the agent for treatment of *H. pylori* infection is highly important since risks of eradication failure by clarithromycin-based regimens were about 7-fold higher (odd ratio, 6.97) in the presence of clarithromycin-resistant strains than for clarithromycin-susceptible strains [15,45].

#### 3.1.4. Tetracycline

Tetracycline is a bacteriostatic agent which reversibly binds to the 30S subunit of *H. pylori* ribosomes containing 16S rRNA, thereby suppressing protein synthesis and bacterial growth [45].

Tetracycline is one of the agents used in bismuth quadruple therapy [7,46]. However, one of the main disadvantages of the bismuth quadruple therapy containing metronidazole and tetracycline has been adverse effects observed in half of the patients [7].

In 2006–2016, primary *H. pylori* resistance rates to tetracycline were low (≤10%) in most countries worldwide [19]. *H. pylori* resistance to tetracycline was associated with single, double and especially simultaneous triple point-mutations within both copies (*rrnA/B* genes) of 16S rRNA [47].

According to the recent studies, there was no increase in *H. pylori* resistance to tetracycline, except in Iran [34] (Table 4).

Very low *H. pylori* resistance rates (0–<4%) were usually observed, and no tetracycline resistance evolution was found in European countries such as Belgium, Bulgaria, France, Russia, and Spain, as well as in many other countries, such as Australia, Chile, China, Colombia, and Taiwan [12,20,21,22,24,25,26,27,29,30,31,32,33] (Boyanova, present study). In Europe, the results can be explained by the decreasing tetracycline (J01A) consumption as a mean 10-year trend [36].

High tetracycline resistance in *H. pylori* (>10%) was rarely found, being detected in consecutive Chinese adult patients (from 1998–1999 to 2016–2017) and Iranian children and adult patients (from 1999–2000 to 2017 to 2019) [33,34].

Importantly, in the French study of Mégraud et al. [22], no tetracycline resistance in *H. pylori* was detected in the untreated and treated patients despite the launch of the single triple capsule of bismuth subcitrate, metronidazole, and tetracycline (Pylera*^®^*).

#### 3.1.5. Levofloxacin

Levofloxacin is a third-generation fluoroquinolone with bactericidal activity, suppressing DNA gyrase of *H. pylori*, since, unlike other bacteria, the species lacks genes for the topoisomerase [10]. The agent is used in eradication regimens such as a levofloxacin-based triple regimen, a sequential therapy regimen, and a concomitant bismuth- and levofloxacin-based therapy [48].

Levofloxacin resistance in *H. pylori* results from mutations in *gyrA* and *gyrB* genes encoding DNA gyrase subunits, and especially in codons 87 and 91 in QRDR (quinolone resistance-determining region) of GyrA [10]. Primary levofloxacin resistance was ≥11–15% in most WHO regions in 2006–2016 [19]. Secondary levofloxacin resistance during the period was high in the Eastern Mediterranean Region and Western Pacific Region (30%) [19].

Recent data showed that in contrast to tetracycline, *H. pylori* resistance to levofloxacin displayed an increase in many European studies, such as those from Belgium, Bulgaria, Italy (two studies), and Russia, [12,20,21,26,41,43] (Boyanova, this study). The rise in fluoroquinolone resistance was also common in non-European countries such as China, Iran, Taiwan (in both untreated and treated patients), and in the overall Western Pacific region [19,32,33,34] (Table 5).

Primary levofloxacin resistance increased in Belgium, Italy, and Taiwan [20,21,32,43] but did not show evolution in other countries such as France, Spain, Chile, and in the study of nine European countries [22,24,25,31,42].

Overall, fluoroquinolone resistance increased in six countries/regions, namely Bulgaria, Italy, Russia, Iran, China and in the Western Pacific region [12,19,26,33,34,41] (Boyanova, this study). In the USA, levofloxacin resistance rates exhibited a significant overall rise from 10.0% in 2000–2001, to approximately 30.0% in 2012–2013, followed by a decrease to <20.0% from 2012–2013 to 2016 [28]. The decrease was associated with the fluoroquinolone restriction policy in the country since 2013 [28].

No significant evolution of *H. pylori* levofloxacin resistance was found in treated patients in France, whereas in Taiwan both primary and post-treatment (2nd line) resistance increased [22,32].

High levofloxacin resistance rates (>30%) were found in consecutive Chinese adults (81.5% in 2016–2017), Iranian children and adults (36.0% in 2017–2019), untreated Italian adults (33.8% in 2015–2019), consecutive Bulgarian patients (30.6% in 2016–2022), and in Taiwan (38.0% in untreated patients in 2019 and >64% in treated patients in 2016–2019), [12,32,33,34,43] (Boyanova, this study).

Levofloxacin resistance can be associated with quinolone (J01M) use. In Europe, a significant association was observed between *H. pylori* resistance to levofloxacin and consumption of second-generation quinolones, such as ciprofloxacin [23]. In Bulgaria, the increase in levofloxacin resistance correlated with higher J01M use (3.9 DDD per 1000 inhabitants per day) compared to that in France, where there was no significant rise in the resistance and the J01M consumption was 1.0 DDD [36]. In France, a decrease (by >25%) in quinolone consumption was detected between 2000 and 2015, and this can explain the lack of increase in levofloxacin resistance rates in the country [22,23].

#### 3.1.6. Double and Multidrug Resistance

In the systematic review and meta-analysis of Savoldi et al. [19] in 2006–2016, primary double resistance to clarithromycin and metronidazole in *H. pylori* varied from <10 to 19% worldwide. Overall secondary resistance to both clarithromycin and metronidazole during the period was 18% in the European Region [19].

Multidrug resistance is simultaneous resistance to ≥3 antibiotics of different categories (classes). *H. pylori* multidrug resistance can result from several simultaneous mutations associated with resistance to different antibiotics, however, efflux pumps, diminished drug uptake and biofilm production, can also be involved [10]. Upregulated expression of TolC homologous genes, such as *hefA* that increases activity of efflux pumps, was observed in multidrug-resistant *H. pylori* strains [40].

According to the recent studies, increasing double resistance to both metronidazole and clarithromycin was found in some countries. The increase was observed in consecutive Bulgarian patients (1.6-fold increase), untreated Taiwanese adults (4.3-fold increase) and in one Italian study of consecutive patients (2.5-fold rise from 2010 to 2013), followed by a plateau in 2014–2016 [12,32,41] (Boyanova, this study). The double metronidazole + clarithromycin resistance showed no evolution in untreated Chilean patients (from 2005 to 2007 to 2015 to 2017), consecutive Colombian adults (from 2009 to 2015), Iranian patients (from 1999 to 2000 to 2011 to 2019), in one of the two Italian articles (from 2009 to 2014 to 2015 to 2019), and in the review study on nine European countries [24,29,30,31,34,43] (Table 6).

Importantly, an increase in overall triple resistance (from 3.7% 2005-2007 to 18.0% in 2015-2017) was observed in strains from untreated children and adults in Chile [31].

Although there were not many recent reports on the evolution of multidrug resistance in *H. pylori*, in our previous review publication on the topic [49], primary multidrug resistance varied from <10% in most of the European countries, to >40% in some countries such as Peru, and overall resistance rates of >23–36% were found in half of the studies. In pediatric patients, multidrug resistance was also found, ranging from 3.8% in Slovenia in 2011–2014 and 6.6% in Bulgaria in 2012–2021, to >20% in untreated children in China [50].

Multidrug resistance in *H. pylori* is a hard challenge to overcome in treatment and although there has been no significant evolution of the resistance in many countries so far, the results emphasized both the importance of laboratory susceptibility testing of the isolates, and the search for new therapeutic drugs and/or regimens.

### 3.2. Factors for Resistance Evolution

*H. pylori* infection is common and affects <20%, up to 90% of the population in different countries [51]. Antibiotic resistance in *H. pylori* has been increasing over time. In the 1980s, clarithromycin resistance rates were from 0% to <9% according to the review article of Lahaie and Gaudreau [52], versus >20% in many patient groups and countries during the last five years.

**Figure 2:**
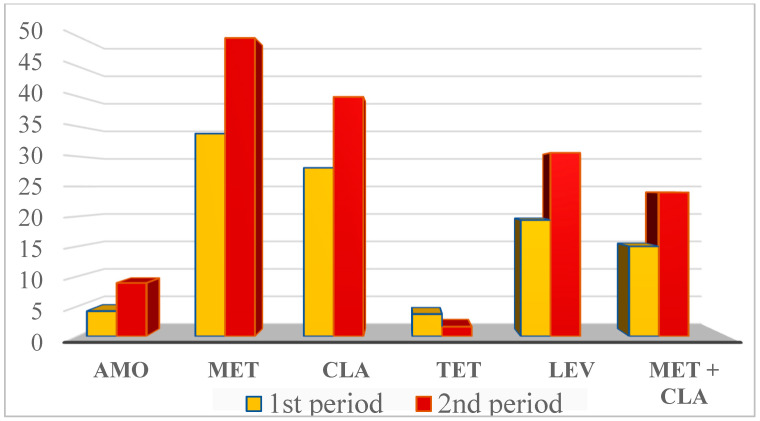
Antibiotic resistance in *H. pylori* from consecutive Bulgarian patients in the first period (2007–2014 for amoxicillin, and 2010–2015 for the other antibiotics) and the second period (2015–2021 for amoxicillin, and 2016–2022 for the other antibiotics) [11,12] (Boyanova, this study).

Important factors of *H. pylori* resistance evolution are the use of currently non-recommended regimens, such as triple clarithromycin-based therapy in regions with high (>15%) resistance to the agent [15,53], national antibiotic consumption, patient compliance and other host factors. High antibiotic consumption in Bulgaria can be associated with an increase in *H. pylori* resistance to all antibacterials, except tetracycline [36] (Figure 2).

In Italy and Spain, primary antibiotic resistance varied according to the patients’ age and sex [42,43]. In Italy, female sex, age (>50 years), body mass index (>25) and smoking were associated with resistance to some of the antibiotics [43].

*H. pylori* virulence factors were also related to resistance of some antibacterials. The systematic review and meta-analysis of Karbalaei et al. [54] showed that less virulent (*vacA* s2m2) strains were associated with lower antibiotic resistance rates, possibly due to lower biofilm production or lower blood flow to the stomach compared to those of more virulent strains. Using antibiofilm agents is a strategy to improve therapy of the infection [55]. Heteroresistance (different susceptibility to specific antibiotics by *H. pylori* subpopulations in the same patient) has also been evaluated [56].

In addition, access to antibiotics over-the-counter (without prescription), mostly in some developing countries, can increase and spread antibiotic resistance [57,58]. Other factors such as azithromycin misuse or overuse since the beginning of the COVID-19 pandemic and migrations from countries with higher resistance rates may also be of importance for *H. pylori* resistance evolution [27,44].

## 4. Conclusions

Knowledge of *H. pylori* resistance evolution to the five most commonly used antibiotics in eradication regimens is necessary to limit treatment failure.

In some countries, such as Bulgaria, Belgium, Iran, and Taiwan, growing *H. pylori* resistance to three or more antibacterial agents has been observed over time, while in other countries, such as France and Spain, resistance to most antibiotics used for *H. pylori* eradication has been stabilized.

The lack of increase in antibiotic resistance and even a decrease in resistance rates were usually related to the decrease in the national antibiotic consumption of the given antibiotic, compliance with the latest guidelines for *H. pylori* infection management and strongly enforced antibiotic policy in some countries, such as France and the USA [22,28].

In 2017, the WHO included clarithromycin-resistant *H. pylori* in high priority bacteria for antibiotic research and development [59]. Current specialists and international guidelines recommend either bismuth- or non-bismuth-based quadruple therapy for 14 days as a first-line treatment for *H. pylori* in regions of high clarithromycin or metronidazole resistance rates [14,15,16,17,18]. In addition, most of the current eradication regimens recommend an extended treatment duration of 14 days. These recommendations are especially important for countries with increasing *H. pylori* antibiotic resistance.

Newer agents, such as vonoprazan, bismuth, in addition to some triple eradication regimens, and evaluation of newer antibiotics with improved stability at low pH and/or increased antibacterial activity, such as delafloxacin, can be considered as well [6,45,48,60,61]. However, regular monitoring of antibiotic resistance rates is utterly important to determine the appropriate eradication regimens in a given country or region.

In addition to the classical susceptibility testing methods, the use of molecular techniques [62], non-invasive tests for detection of resistance or adjuvants, such as antibiofilm substances [49] and nanoparticles [63], as well as the development of vaccines, are strategies to control both *H. pylori* infection and antibiotic resistance.

Most importantly, stabilizing and even reducing *H. pylori* antibiotic resistance, which has already been reported in some studies, is an important and achievable goal for all countries, provided that antibiotic overuse and misuse are reduced, antibiotic policy is strictly followed, and the recent guidelines are complied with in practice.

## Figures and Tables

**Table 1:** Evolution of *H. pylori* resistance to amoxicillin according to some recent data.

Continent/ Country	Patients	Method	Breakpoints	No. of Patients	Resistance (% in Years)	Resistance Evolution	Reference
**Asia**							
China	Consecutive adults	E test, PCR	Specified	23 in 1998–1999, 50 in 2002–2004, 27 in 2016–2017	4.3 in 1998–1999, 2.0 in 2002–2004, 25.9 in 2016–2017	Increase	[33]
Iran	Children and adults	DDM, ADM	NA (review)	3619 in 1999–2019	9.0 in 1999–2000, 14.0 in 2011–2016, 36.0 in 2017–2019	Increase	[34]
Taiwan	Untreated adults	E test	Specified	1369 in 2013–2019	≤0.6 in 2013–2014, ≤1.0 in 2015–2019	No	[32]
Taiwan	Adults, treated (2nd-line treatment)	E test	Specified	196 in 2013–2019	0.0 in 2013–2019	No	[32]
Taiwan	Adults, treated (3rd-line treatment)	E test	Specified	184 in 2013–2019	0.0 in 2013–2014, 2.4 in 2015, 5.9 in 2016, 0.0 in 2017–2018, 5.6 in 2019	No	[32]
Vietnam	Untreated adults and other data	E test, *pbp1A* sequencing	NA	308 in 2019/2021 and previous data	≤1.1 in 2013–2015, 10.4 in 2016, 15.0 in 2019, 25.7% in 2020	Increase	[35]
**Australia/** **Oceania**							
Australia	Consecutive adults (mainly untreated)	E test	EUCAST	907 in 1998–2007, 566 in 2008–2017	0.2 in 1998–2007, 0.0 in 2008–2017	No	[27]
**Europe**							
Belgium	Untreated patients, children and adults	DDM, E test	EUCAST	1001 in 2008–2009, 438 in 2016	0.0 in 2008–2009, 0.0 in 2016	No	[20,21]
Bulgaria	Consecutive, children and adults	E test	EUCAST	237 in 2007–2014, 237 in 2015–2021	4.2 in 2007–2014, 8.9 in 2015–2021	Increase	[11]
France	Untreated adults	E test, DDM	EUCAST	266 in 2014, 231 in 2016, 244 in 2018	0.7 in 2014, 0.9 in 2016, 0.0 in 2018	No	[22]
France	Treated adults	E test, DDM	EUCAST	115 in 2014, 125 in 2016, 110 in 2018	0.0 in 2014, 0.8 in 2016, 0.0 in 2018	No	[22]
Russia	Consecutive adult patients	ADM	EUCAST	133 in 2009–2010, 143 in 2015–2017	4.5 in 2009–2010, 1.4 in 2015–2017	No	[26]
Nine European countries *	Untreated patients	NA (review)	NA (review)	2000 in 2013–2016, 1250 in 2017–2020	0.3 in 2013–2016, 0.4 in 2017–2020	No	[24]
**South America**							
Chile	Untreated children and adults	DDM	Specified	299 in 2005–2007, 72 in 2015–2017	2.0 in 2005–2007, 4.2 in 2015–2017	No	[31]
Colombia	Consecutive adults	E test	CLSI, EUCAST	106 in 2009, 61 in 2015	1.9 in 2009, 0.0 in 2015	No	[29,30]

NA—not available or not appropriate, specified—specified by the authors, methods—DDM-disk diffusion method, ADM—agar dilution method. * Nine European countries—Italy, Spain, Norway, Greece, Slovenia, Israel, Russia, France, Ireland.

**Table 2:** Evolution of *H. pylori* resistance to metronidazole according to some recent data.

Continent/Country	Patients	Method	Breakpoints	No. of Patients	Resistance (% in Years)	Resistance Evolution	Reference
**Asia**							
China	Consecutive adults	E test, PCR	Specified	23 in 1998–1999, 50 in 2002–2004, 27 in 2016–2017	87.0 in 1998–1999, 66.0 in 2002–2004, 92.6 in 2016–2017	No	[33]
Iran	Children and adults	DDM, ADM	NA (review)	4330 in 1999–2019	67.0 in 1999–2000, 52.0 in 2011–2016, 66.0 in 2017–2019	No	[34]
Taiwan	Untreated adults	E test	Specified	1369 in 2013–2019	25.6 in 2013, >29.0 in 2015–2018, 42.3 in 2019	Increase	[32]
Taiwan	Adults, treated (2nd-line treatment)	E test	Specified	196 in 2013–2019	≤50.0 in 2013–2016, >70.0 in 2017–2019	Increase	[32]
Taiwan	Adults, treated (3rd-line treatment)	E test	Specified	184 in 2013–2019	44.4 in 2013, <53.0 in 2014–2015 to 83.3 in 2019	Increase	[32]
**Australia/Oceania**							
Australia	Consecutive adults (mainly untreated)	E test	EUCAST	907 in 1998–2007, 566 in 2008–2017	32.3 (1998–2007), 39.9 (2008–2017)	No	[27]
**Europe**							
Belgium	Untreated children and adults	DDM, E test	EUCAST	1001 in 2008–2009, 438 in 2016	28.0 in 2008–2009, 40.0 in 2016	Increase	[20,21]
Bulgaria	Consecutive adults and children	E test, DDM	EUCAST	299 in 2010–2015, 183 in 2016–2022	33.8 in 2010–2015, 49.7 in 2016–2022	Increase	[12], Boyanova, this study
France	Untreated adults	E test, DDM	EUCAST	266 in 2014, 231 in 2016, 244 in 2018	45.9 in 2014, 52.4 in 2016, 58.6 in 2018	Increase	[22]
France	Treated adults	E test, DDM	EUCAST	115 in 2014, 125 in 2016, 110 in 2018	78.3 in 2014, 80.6 in 2016, 87.3 in 2018	Slight increase	[22]
Italy	Consecutive untreated adults	E test	EUCAST	907 in 2009–2014, 739 in 2015–2019	33.3 in 2009–2014, 33.6 in 2015–2019	No	[43]
Italy	Consecutive patients	E test	EUCAST	1424 in 2010–2016	33.6 in 2010, 45.3 in 2013, 40.2 in 2016	Increase in 2010/2013, plateau in 2014–2016	[41]
Russia	Consecutive adult patients	ADM	EUCAST	133 in 2009–2010, 143 in 2015–2017	3.8 in 2009–2010, 23.8 in 2015–2017	Increase	[26]
Spain	Children untreated and treated	E test	EUCAST	27 in in 2014–2015, 24 in in 2016–2017, 29 in 2018–2019	Around 30 in 2014–2015 and 2018–2019	No	[25]
Spain	Untreated patients, mostly adults	E test	EUCAST	254 in 2004–2005, 233 in 2015–2016	45.0 in 2004, 41.0 in 2005, 30.0 in 2015, 34.0 in 2016	Decrease	[42]
Nine European countries *	Untreated patients	NA (review)	NA (review)	1733 in 2013–2016, 1139 in 2017–2020	33.0 in 2013–2016, 24.5 in 2017–2020	Decrease	[24]
**South America**							
Chile	Untreated children and adults	DDM	Specified	364 in 2005–2007, 72 in 2015–2017	55.5 in 2005–2007, 37.5 in 2015–2017	Decrease	[31]
Colombia	Consecutive adults	E test	CLSI, EUCAST	106 in 2009, 61 in 2015	82.0 in 2009, 78.7 in 2015	No	[29,30]

NA—not available or not appropriate, specified—specified by the authors, methods—DDM-disk diffusion method, ADM—agar dilution method. * Nine European countries (Hp-EuReg)—Italy, Spain, Norway, Greece, Slovenia, Israel, Russia, France, Ireland.

**Table 3:** Evolution of *H. pylori* resistance to clarithromycin in different countries/regions according to some recent data.

Continent/ Country	Patients	Method	Breakpoints	No. of Patients	Resistance (% in years)	Resistance Evolution	Reference
**Asia**							
China	Consecutive adults	E test, PCR	Specified	23 in 1998–1999, 50 in 2002–2004, 27 in 2016–2017	39.1 in 1998–1999, 14.0 in 2002–2004, 55.6 in 2016–2017	No	[33]
Iran	Children and adults	DDM, ADM	NA (review)	5145 in 1999–2019	9.0 in 1999–2000, 21.0 in 2011–2016, 36.0 in 2017–2019	Increase	[34]
Taiwan	Untreated adults	E test	Specified	1369 in 2013–2019	<14.0 in 2013–2014, 20.4 in 2019	Increase	[32]
Taiwan	Treated adults (2nd-line treatment)	E test	Specified	196 in 2013–2019	70.3 in 2013, <70.0 in 2014 and 2017–2018, 80.6 in 2019	No	[32]
Taiwan	Treated adults (3rd-line treatment)	E test	Specified	184 in 2013–2019	<77.0 in 2013–2014, 80.5–92.3 in 2015–2017, 64.0 in 2018, 83.3 in 2019	No	[32]
South-East Asia	Overall patients	NA (review)	NA (review)	1830 in 2006–2016	13 in 2006–2008, 21 in 2012–2016	Increase	[19]
**Australia/Oceania**							
Australia	Consecutive adults (mainly untreated)	E test	EUCAST	907 in 1998–2007, 566 in 2008–2017	16.0 (1998–2007), 21.2 (2008–2017)	Increase	[27]
**Europe**							
Belgium	Untreated children and adults	DDM, E test	EUCAST	1001 in 2008–2009, 438 in 2016	10.5 in 2008–2009, 18.0 in 2016	Increase	[20,21]
Bulgaria	Consecutive adults and children	E test, DDM	EUCAST	299 in 2010–2015, 183 in 2016–2022	28.1 in 2010–2015, 39.9 in 2016–2022	Increase	[12], Boyanova, this study
France	Untreated adults	E test, DDM, PCR	EUCAST	266 in 2014, 231 in 2016, 244 in 2018	22.2 in 2014, 20.3 in 2016, 20.9 in 2018	No	[22]
France	Treated adults	E test, DDM, PCR	EUCAST	115 in 2014, 125 2016, 110 in 2018	73.9 in 2014, 59.7 in 2016, 56.4 in 2018	Decrease	[22]
Italy	Consecutive untreated adults	E test	EUCAST	907 in 2009–2014, 739 in 2015–2019	30.2 in 2009–2014, 37.8 in 2015–2019	Increase	[43]
Italy	Consecutive patients	E test	EUCAST	1424 in 2010–2016	19.0 in 2010, 35.6 in 2013, 35.9 in 2016	Increase (2010/13), plateau in 2014/16	[41]
Russia	Consecutive adult patients	ADM	EUCAST	133 in 2009–2010, 143 in 2015–2017	5.3 in 2009–2010, 6.3 in 2015–2017	No	[26]
Spain	Children untreated and treated	E test	EUCAST	80 in 2014–2019	Around 50.0 in 2014–2015 and 2018–2019	No	[25]
Spain	Consecutive patients, mostly adults	E test	EUCAST	253 in in 2004–2005, 233 in in 2015–2016	21.0 in 2004, 20.0 in 2005, 23.0 in 2015, 22.0 in 2016	No	[42]
Nine European countries *	Untreated patients	NA (review)	NA (review)	1724 in 2013–2016, 1150 in 2017–2020	25.0 in 2013–2016, 20.0 in 2017–2020	No	[24]
**South America**							
Chile	Untreated children and adults	DDM	Specified	333 in 2005–2007, 72 in 2015–2017	22.5 in 2005–2007, 29.2 in 2015–2017	No	[31]
Colombia	Consecutive adults	E test, sequencing	CLSI, EUCAST	106 in 2009, 61 in 2015	3.8 in 2009, 8.2 in 2015	No	[29,30]

NA—not available or not appropriate, specified—specified by the authors, methods—DDM-disk diffusion method, ADM—agar dilution method. * Nine European countries—Italy, Spain, Norway, Greece, Slovenia, Israel, Russia, France, Ireland.

**Table 4:** Evolution of *H. pylori* resistance to tetracycline according to some recent data.

Continent/Country	Patients	Method	Breakpoints	No. of Patients	Resistance (% in Years)	Resistance Evolution	Reference
**Asia**							
China	Consecutive adults	E test, PCR	Specified	23 in 1998–1999, 50 in 2002–2004, 27 in 2016–2017	13.0 in 1998–1999, 14.0 in 2002–2004, 18.5 in 2016–2017	No	[33]
Iran	Children and adults	DDM, ADM	NA (review)	3061 in 1999–2019	3.0 in 1999–2000, 12.0 in 2011–2016, 18.0 in 2017–2019	Increase	[34]
Taiwan	Untreated adults	E test	Specified	1369 in 2013–2019	0.0 in 2013–2014, 0.8 in 2015, 0.0 in 2016–2019	No	[32]
Taiwan	Treated adults (2nd-line treatment)	E test	Specified	196 in 2013–2019	0.0 except for 1.7 in 2014 and 7.1 in 2016	No	[32]
Taiwan	Treated adults (3rd-line treatment)	E test	Specified	184 in 2013–2019	11.1 in 2013, 0.0–7.7 in 2014–2017, 0.0 in 2018–2019	No	[32]
**Australia/Oceania**							
Australia	Consecutive adults (mainly untreated)	E test	EUCAST	907 in 1998–2007, 566 in 2008–2017	0.2 (1998–2007), 0.5 (2008–2017)	No	[27]
**Europe**							
Belgium	Untreated patients, children and adults	DDM, E test	EUCAST	1001 in 2008–2009, 438 in 2016	0.0 in 2008–2009, 0.0 in 2016	No	[20,21]
Bulgaria	Consecutive adults and children	E test, DDM	EUCAST	134 in 2010–2015, 183 in 2016–2022	3.7 in 2010–2015, 1.6 in 2016–2022	No	[12], Boyanova, this study
France	Untreated adults	E test, DDM	EUCAST	266 in 2014, 231 in 2016, 244 in 2018	0.0 in 2014, 0.0 in 2016, 0.0 in 2018	No	[22]
France	Treated adults	E test, DDM	EUCAST	115 in 2014, 125 in 2016, 110 in 2018	0.0 in 2014, 0.0 in 2016, 0.0 in 2018	No	[22]
Russia	Consecutive adult patients	ADM	EUCAST	133 in 2009–2010, 143 in 2015–2017	0.0 in 2009–2010, 0.7 in 2015–2017	No	[26]
Spain	Children untreated and treated	E test	EUCAST	80 in 2014–2019	0 in 2014–2015 and 2018–2019	No	[25]
Nine European countries *	Untreated patients	NA (review)	NA (review)	2000 in 2013–2016, 1250 in 2017–2020	0.2 in 2013–2016, 0.08 in 2017–2020	No	[24]
**South America**							
Chile	Untreated children and adults	DDM	Specified	311 in 2005–2007, 72 in 2015–2017	1.0 in 2005–2007, 1.4 in 2015–2017	No	[31]
Colombia	Consecutive adults	E test	CLSI, EUCAST	106 in 2009, 61 in 2015	0.0 in 2009, 0.0 in 2015	No	[29,30]

NA—not available or not appropriate, specified—specified by the authors, methods—DDM-disk diffusion method, ADM—agar dilution method. * Nine European countries (Hp-EuReg)—Italy, Spain, Norway, Greece, Slovenia, Israel, Russia, France, Ireland.

**Table 5:** Evolution of *H. pylori* resistance to fluoroquinolones according to some recent data.

Continent/ Country	Patients	Method	Breakpoints	No. of Patients	Resistance (% in Years)	Resistance Evolution	Reference
**Asia**							
China	Consecutive adults	E test, PCR	Specified	23 in 1998–1999, 50 in 2002–2004, 27 in 2016–2017	47.8 in 1998–1999, 46.0 in 2002–2004, 81.5 in 2016–2017	Increase	[33]
Iran (ciprofloxacin)	Children and adults	DDM, ADM	NA (review)	2046 in 1999–2019	15.0 in 1999–2000, 18.0 in 2011–2016, 36.0 in 2017–2019	Increase	[34]
Taiwan	Untreated adults	E test	Specified	1369 in 2013–2019	<24.0 in 2013–2015, 29.1–32.4 in 2016–2018, 38.8 in 2019	Increase	[32]
Taiwan	Treated adults (2nd-line treatment)	E test	Specified	196 in 2013–2019	<38.0 in 2013–2015, 51.6–64.7 in 2016–2019	Increase	[32]
Taiwan	Treated adults (3rd-line treatment)	E test	Specified	184 in 2013–2019	72.2 in 2013, >94.0 in 2016 and 2017, 76.0 in 2018, 83.3 in 2019	No	[32]
Western Pacific region	Overall patients	NA (review)	NA (review)	28946 in 2006–2016	12.0 in 2006–2008, 31.0 in 2012–2016	Increase	[19]
**Europe**							
Belgium	Untreated patients, children and adults	DDM, E test	EUCAST	1001 in 2008–2009, 438 in 2016	12.4 in 2008–2009, 22.8 in 2016	Increase	[20,21]
Bulgaria	Consecutive adults and children	E test, DDM	EUCAST	299 in 2010–2015, 183 in 2016–2022	19.4 in 2010–2015, 30.6 in 2016–2022	Increase	[12], Boyanova, this study
France	Untreated adults	E test, DDM	EUCAST	266 in 2014, 231 in 2016, 244 in 2018	15.4 in 2014, 14.7 in 2016, 17.6 in 2018	No	[22]
France	Treated adults	E test, DDM	EUCAST	115 in 2014, 125 in 2016, 110 in 2018	14.8 in 2014, 23.4 in 2016, 22.7 in 2018	Slight increase	[22]
Italy	Consecutive untreated adults	E test	EUCAST	907 in 2009–2014, 739 in 2015–2019	25.6 in 2009–2014, 33.8 in 2015–2019	Increase	[43]
Italy	Consecutive patients	E test	EUCAST	1424 in 2010–2016	19.0 in 2010, 29.7 in 2013, 29.3 in 2016	Increase (2010/13), plateau in 2013/16	[41]
Russia	Consecutive adult patients	ADM	EUCAST	133 in 2009–2010, 143 2015–2017	8.3 in 2009–2010, 24.5 in 2015–2017	Increase	[26]
Spain	Children untreated and treated	E test	EUCAST	80 in 2014–2019	Around 10.0 in 2014–2015 and 2018–2019	No	[25]
Spain	Consecutive patients, mostly adults	E test	EUCAST	212 in 2011–2012, 233 in 2015–2016	15.0 in 2011, 17.0 in 2012, 22.0 in 2015, 17.0 in 2016	No	[42]
Nine European countries *	Untreated patients	NA (review)	NA (review)	1717 in 2013–2016, 1155 in 2017–2020	20.5 in 2013–2016, 18.0 in 2017–2020	No	[24]
**North America**							
USA	Consecutive patients	E test	Specified	800 in 2000 to 2016	<10.0 in 2000–2001, around 30.0 in 2012-2013, <20.0 in 2016	Overall increase, decrease since 2012/13	[28]
**South America**							
Chile	Untreated children and adults	DDM	Specified	321 in 2005–2007, 72 in 2015–2017	15.3 in 2005–2007, 20.8 in 2015–2017	No	[31]

NA—not available or not appropriate. In Iran, ciprofloxacin was tested, in all other countries, susceptibility to levofloxacin was evaluated. Methods—DDM-disk diffusion method, ADM—agar dilution method. * Nine European countries—Italy, Spain, Norway, Greece, Slovenia, Israel, Russia, France, Ireland.

**Table 6:** Evolution of double and multidrug resistance according to some recent data.

Resistance to	Continent/ Country	Patients	Method	Breakpoints	No. of Patients	Resistance (% in Years)	Resistance Evolution	Reference
**MET + CLA**	**Asia**							
	Iran	Children and adults	DDM, ADM	NA (review)	1562 in 1999–2019	15.0 in 1999–2000, 17.0 in 2011–2016, 17.0 in 2017–2019	No	[34]
	Taiwan	Untreated adults	E test	Specified	1369 in 2013–2019	2.4 in 2013, 5.1–8.4 in 2014–2018, 10.4 in 2019	Increase	[32]
	**Europe**							
	Bulgaria	Consecutive adults and children	E test, DDM	EUCAST	299 in 2010–2015, 183 in 2016–2022	15.0 in 2010–2015, 24.0 in 2016–2022	Increase	[12], Boyanova, this study
	Italy	Consecutive untreated adults	E test	EUCAST	907 in 2009–2014, 739 in 2015–2019	18.9 in 2009–2014, 20.7 in 2015–2019	No	[43]
	Italy	Consecutive patients	E test	EUCAST	1424 in 2010–2016	11.4 in 2010, 28.2 in 2013, 21.9 in 2016	Increase (2010–2013), plateau in 2014–2016	[41]
	Nine European countries *	Untreated patients	NA (review)	NA (review)	1728 in 2013–2016, 1145 in 2017–2020	14.0 in 2013–2016, 11.0 in 2017–2020	No	[24]
	**South America**							
	Chile	Untreated children and adults	DDM	Specified	271 in 2005–2007, 72 in 2015–2017	12.2 in 2005–2007, 18.0 in 2015–2017	No	[31]
	Colombia	Consecutive adults	E test	CLSI, EUCAST	106 in 2009, 61 in 2015	3.8 in 2009, 8.2 in 2015	No	[29,30]
**MDR triple**	**Europe**							
	Italy	Consecutive untreated adults	E test	EUCAST	907 in 2009–2014, 739 in 2015–2019	10.4 in 2009–2014, 12.6 in 2015–2019	No	[43]
	Nine European countries *	Untreated patients	NA (review)	NA (review)	1722 in 2013–2016, 1133 in 2017–2020	7.2 in 2013–2016, 4.5 in 2017–2020	No	[24]
	**South America**							
	Chile	Untreated children and adults	DDM	Specified	271 in 2005–2007, 72 in 2015–2017	3.7 in 2005–2007, 18.0 in 2015–2017	Increase	[31]
**MDR quadruple**	**South America**							
	Chile	Untreated children and adults	DDM	Specified	271 in 2005–2007, 72 in 2015–2017	0.4 in 2005–2007, 2.8 in 2015–2017	No	[31]

MET—metronidazole, CLA—clarithromycin, NA—not available or not appropriate, MDR—multidrug resistance, methods—DDM-disk diffusion method. * Nine European countries—Italy, Spain, Norway, Greece, Slovenia, Israel, Russia, France, Ireland.

## Data Availability

Data are contained within the review.
